# Editorial Comment: Effects of mesh surgery on sexual function in pelvic prolapse and urinary incontinence

**DOI:** 10.1590/S1677-5538.IBJU.2019.0618.1

**Published:** 2020-11-18

**Authors:** Cássio Luís Zanettini Riccetto

**Affiliations:** 1 Universidade Estadual de Campinas Faculdade de Ciências Médicas Divisão de Urologia CampinasSP Brasil Divisão de Urologia - Faculdade de Ciências Médicas - Universidade Estadual de Campinas – UNICAMP, Campinas, SP, Brasil

## COMMENT

In this issue of the Int Braz J Urol, Sukgen and colleagues (1) presented the evolution of the Female Sexual function Index (FSFI) in a prospective series of 72 women who underwent correction of pelvic organ prolapse (POP), eventually associated to stress urinary incontinence (SUI), using a four-arm anterior mesh implant (Betamix POP4®, Betatech Medical, Turkey) with a transobturator fixation. The study only included patients with POP stage 3 or 4 according to the Pelvic Organ Prolapse Quantification system (POP-Q). Procedures varied based on the vaginal compartments involved and SUI concomitance. This study concluded that POP surgery using mesh implants was associated with a significant improvement in patient sexual function over one year follow-up.

The human sexual response, and women's in particular, is a multidimensional phenomenon (2, 3). In patients with POP, association with urinary incontinence, the impairment of self-image and the sensation of vaginal enlargement and laxity makes the assessment of the impact of any treatment in sexual function quite complex. Although FSFI has been culturally adapted to several languages and represent an alternative for a comprehensive evaluation of sexual function (4), it does not specifically assess vaginal symptoms (5), which is very relevant after POP surgery.

In Sukgen and colleagues study, a prosthesis with dual function was used, since, in patients with associated urinary incontinence, it was adjusted in anterior vaginal wall in order to reach the proximal aspect of the urethra. However, nowadays we are experiencing a trend for the use synthetic transvaginal implants only for repositioning the vaginal apex rather than more extensive prostheses, such as the one used by Sukgen and colleagues, and combine it with a midurehral sling implanted through another suburethral incision in case of concomitant SUI. The possible impact of this trend in sexual function is still to be stablished (1).

The relevance of urinary incontinence in female sexual function has already been extensively investigated (6, 7), thus, greater influence is given to urge urinary incontinence rather than stress urinary incontinence. As POP can be associated with both incontinence types, treatment can thereby improve sexual function.

As the anterior arms of the prosthesis used by the authors act as a midurethral sling, it is relevant to comment about the influence of synthetic sling implant on sexual function. This issue was recently assessed in a meta-analysis (8), which concluded that although overall sexual function remained the same or improved for most women, improvements in orgasmic function were only observed in one third of cases after a midurethral sling procedure. The possible deterioration of orgasm in patients who underwent midurethral slings could be due to a denervation of the periurethral area resulting from the local dissection. In this sense, in 2017, Arslan and colleagues proposed a modification in the transobturatory sling technique, based on a minimal paraurethral dissection, in order to minimize the risk of sexual dysfunction (9). In another study from Tepe and colleagues, sexual function was studied in a group of patients who underwent transobturator sling (TOT) plus vaginal hysterectomy versus Kelly colpoplasty plus vaginal hysterectomy for the treatment of urinary incontinence and uterine prolapse. Despite the potential effect of hysterectomy on sexual function, and the greater efficacy of TOT in the treatment of urinary incontinence, it was notable that the rate of the patients who had FSFI scores greater than 25, which indicated a better sexual function, was significantly higher in the group who underwent Kelly colpoplasty than TOT (10).

POP is considered more relevant than urinary and fecal incontinence for sexual aversion, sexual inactivity and general sexual dissatisfaction (11). However, the effect of surgery for prolapse and incontinence on sexual function is difficult to assess in publications, as randomized studies are scarce, surgical techniques and outcome measure are very variable, and there is a lack of long-term follow-up. In addition, sexual dysfunction is usually a secondary outcome. In general, it is speculated that POP treatment through abdominal, laparoscopic or robotic approaches, even with a mesh implant, leads to lower frequency of sexual dysfunction compared to trasvaginal POP correction, due to the potential deleterious effect of the vaginal incision and dissection (12,13). In Sukgen and colleagues study, they described a progressive improvement of the FSFI, with a low incidence of vaginal exposure, which can be attributed to the age of the patients, mostly in the menacme or climacteric, and to the limited follow-up period, which extended for at most 12 months.

Dyspareunia and chronic pelvic pain are identified as the most serious adverse effects of transvaginal prostheses, with a harmful effect on sexual function. In a recent meta-analysis, Liao and colleagues found that the rate of de novo dyspareunia was 9.9% versus 9.0% in patients who underwent correction using mesh versus native tissue, respectively, with no significant differences in score PISQ-12 (Pelvic Organ Prolapse / Urinary Incontinence Sexual Questionnaire) (14). In Sukgen and colleagues study, despite displaying higher rates of dyspareunia (15.2%) it did not prevent the improvement of the global FSFI score, which also corroborates the for the complexity nature of female sexuality.

In conclusion, patients’ expectations regarding their sexuality are changing intensely. As the demand for POP and incontinence treatments trend to increase in parallel, studies like Sukgen and colleagues’ one can stimulate relevant discussions in the scientific community about this relevant aspect of quality of life.
